# High prevalence of HIV, chlamydia and gonorrhoea among men who have sex with men and transgender women attending trusted community centres in Abuja and Lagos, Nigeria

**DOI:** 10.7448/IAS.19.1.21270

**Published:** 2016-12-07

**Authors:** Babajide Keshinro, Trevor A Crowell, Rebecca G Nowak, Sylvia Adebajo, Sheila Peel, Charlotte A Gaydos, Cristina Rodriguez-Hart, Stefan D Baral, Melissa J Walsh, Ogbonnaya S Njoku, Sunday Odeyemi, Teclaire Ngo-Ndomb, William A Blattner, Merlin L Robb, Manhattan E Charurat, Julie Ake, for the TRUST/RV368 Study Group

**Affiliations:** 1 Department of Defense Walter Reed Program-Nigeria, Abuja, Nigeria; 2 Walter Reed Army Institute of Research, U.S. Military HIV Research Program, Silver Spring, MD, USA; 3 Henry M. Jackson Foundation for the Advancement of Military Medicine, Bethesda, MD, USA; 4 Institute of Human Virology, University of Maryland, Baltimore, MD, USA; 5 Population Council Nigeria, Abuja, Nigeria; 6 Division of Infectious Diseases, Johns Hopkins University School of Medicine, Baltimore, MD, USA; 7 Department of Epidemiology, Johns Hopkins Bloomberg School of Public Health, Baltimore, MD, USA; 8 Institute of Human Virology Nigeria, Abuja, Nigeria

**Keywords:** HIV, chlamydia, gonorrhoea, prevalence, MSM, Nigeria

## Abstract

**Introduction:**

Sexually transmitted infection (STI) and HIV prevalence have been reported to be higher amongst men who have sex with men (MSM) in Nigeria than in the general population. The objective of this study was to characterize the prevalence of HIV, chlamydia and gonorrhoea in this population using laboratory-based universal testing.

**Methods:**

TRUST/RV368 represents a cohort of MSM and transgender women (TGW) recruited at trusted community centres in Abuja and Lagos, Nigeria, using respondent-driven sampling (RDS). Participants undergo a structured comprehensive assessment of HIV-related risks and screening for anorectal and urogenital *Chlamydia trachomatis* and *Neisseria gonorrhoeae*, and HIV. Crude and RDS-weighted prevalence estimates with 95% confidence intervals (CIs) were calculated. Log-binomial regression was used to explore factors associated with prevalent HIV infection and STIs.

**Results:**

From March 2013 to January 2016, 862 MSM and TGW (316 in Lagos and 546 in Abuja) underwent screening for HIV, chlamydia and gonorrhoea at study enrolment. Participants’ median age was 24 years [interquartile range (IQR) 21–27]. One-third (34.2%) were identified as gay/homosexual and 65.2% as bisexual. The overall prevalence of HIV was 54.9%. After adjusting for the RDS recruitment method, HIV prevalence in Abuja was 43.5% (95% CI 37.3–49.6%) and in Lagos was 65.6% (95% CI 54.7–76.5%). The RDS-weighted prevalence of chlamydia was 17.0% (95% CI 11.8–22.3%) in Abuja and 18.3% (95% CI 11.1–25.4%) in Lagos. Chlamydia infection was detected only at the anorectal site in 70.2% of cases. The RDS-weighted prevalence of gonorrhoea was 19.1% (95% CI 14.6–23.5%) in Abuja and 25.8% (95% CI 17.1–34.6%) in Lagos. Overall, 84.2% of gonorrhoea cases presented with anorectal infection only. Over 95% of STI cases were asymptomatic. In a multivariable model, increased risk for chlamydia/gonorrhoea was associated with younger age, gay/homosexual sexual orientation and higher number of partners for receptive anal sex. HIV infection was associated with older age, female gender identity and number of partners for receptive anal sex.

**Conclusions:**

There is a high burden of infection with HIV and asymptomatic chlamydia and gonorrhoea among MSM and TGW in Nigeria. Most cases would have been missed without anorectal screening. Interventions are needed to target this population for appropriate STI screening and management beginning at a young age.

## Introduction

Globally, men who have sex with men (MSM) and transgender women (TGW) bear a disproportionately large burden of HIV infection as compared to individuals with other risk factors for HIV acquisition [1–3]. In sub-Saharan Africa, heterosexual transmission is the primary mode of transmission of HIV, but there is an increasing recognition of the role of MSM in transmission dynamics with risks of onward HIV transmission to both male and female sexual partners [4]. However, available services are largely targeted at prevention only of heterosexual transmission [5]. In many of the countries across sub-Saharan Africa, sexual intercourse between people of the same sex is criminalized, including in Nigeria [6]. In addition, MSM and TGW face significant social stigma and internalized homophobia that may manifest as barriers to the uptake of routine services including screening for HIV and other sexually transmitted infections (STIs) [7–13].

In Nigeria, the 2010 Integrated Behavioural and Biological Surveillance Survey (IBBSS), a nationwide HIV and STI prevalence survey of transport workers, female sex workers, MSM, military personnel and police personnel, demonstrated an average HIV prevalence among MSM of 17.2% (range 15.8–37.6%) [14], while another survey of MSM using symptom-based screening for STI with urethral swabs yielded a 4.2% prevalence of gonorrhoea and a zero percent prevalence of chlamydia amongst MSM in Lagos [15]. In contrast to the symptom-based urethral STI screening methodology used in the latter survey, the use of universal (regardless of symptoms) laboratory-based screening has yielded higher prevalence estimates of STIs amongst MSM in several sub-Saharan countries [16–19]. Data characterizing the burden of HIV and STIs amongst MSM in Nigeria using a universal screening methodology are needed in order to guide screening and management in this vulnerable population.

The objective of this study was to characterize the prevalence of HIV, chlamydia and gonorrhoea among highly marginalized populations of MSM and TGW in two major Nigerian cities – Abuja and Lagos – using universal screening methods, ultimately informing screening and management guidelines for MSM and TGW to combat the epidemic of HIV and other STIs. This study also explored risk factors associated with these infections in the MSM and TGW populations.

## Methods

The RV368/TRUST cohort is a prospective observational study of MSM and TGW in Abuja and Lagos, Nigeria, at MSM friendly health centres. The study was approved by the University of Maryland Baltimore Institutional Review Board (IRB); the Federal Capital Territory Health Research Ethics Committee, Abuja; Walter Reed Army Institute of Research IRB; and the Ministry of Defence Health Research Ethics Committee, Nigeria. All study participants provided voluntary informed consent.

### Study population

Participants were recruited using respondent-driven sampling (RDS) according to previously described methods [20–22]. Briefly, study staff recruited an initial small group of participants (“seeds”) who had a variety of different sociodemographic characteristics and lived in a variety of neighbourhoods across Abuja and Lagos, Nigeria. Each seed was provided three coupons to distribute to other potential participants in the MSM community. Each subsequent participant who was recruited into the study was given another three coupons and recruitment continued in this manner. This form of recruiting may have reached a wider and more diverse population, including a more marginalized group of study participants [21].

To be enrolled, each participant had to be an adult (age 16 years or older at the Abuja site and 18 years or older at the Lagos site) who was assigned a male gender at birth and reported receptive or insertive anal intercourse at least once in the previous 12 months. The participant must also have presented a valid RDS coupon.

Upon enrolment, each participant underwent screening for HIV and STIs. Demographic and behavioural data were collected using a structured interview and questionnaire administered by study staff. These data included information about occupation, HIV risk factors and sexual behaviours. A complete medical examination with documentation of medical history was performed by a study physician. The physician asked each participant about the presence of specific symptoms of STIs such as fevers/sweats, thrush/mouth sores, rash, genital discharge, genital/rectal pain and painful urination.

Participants who enrolled in the cohort between 20 March 2013 and 18 January 2016 with documented screening results for HIV, chlamydia and gonorrhoea were included in this analysis.

### Testing for HIV and other sexually transmitted infections

Finger stick blood samples were collected to ascertain HIV infection status using a parallel algorithm of Determine^®^ (Alere, Watham, MA, USA) and Uni-gold^®^ (Trinity Biotech, Co-Wicklow, Ireland). Voided urine and rectal swabs were collected and tested for *Chlamydia trachomatis* and *Neisseria gonorrhoeae* using the ultra-sensitive Aptima Combo 2^®^ assay (Hologic, Bedford, MA, USA). All assays were performed according to package inserts.

### Data analysis

Data from the first visit when participants were screened for HIV and STIs were used for this analysis. Pre-selected demographic characteristics and behavioural risk factors of interest were assigned to mutually exclusive categories. A missing indicator variable was included for cases in which data were unavailable, including participant refusal to answer specific questions. Analyses were stratified by clinical care site and by HIV status. Crude and RDS-weighted prevalence estimates with 95% confidence intervals (CIs) were calculated for each demographic characteristic and STI. This method uses personal network size and recruitment pattern data to account for the non-random recruitment method and generate unbiased population estimates [23,24]. The RDS-weighted prevalence estimates and their standard errors were used to calculate a *z*-score and its associated *p*-value for comparisons between clinical care sites. Data from the two clinical care sites were pooled, and log-binomial regression was used in univariable and multivariable models to explore factors associated with prevalent HIV and STIs. All analyses were performed using Stata 13.0 (StataCorp LP, College Station, TX, USA).

## Results

### Demographic and behavioural characteristics

Participants were recruited from an initial group of 10 seeds, including five seeds in Abuja with up to 27 waves of accrual and five seeds in Lagos with up to 24 waves of accrual. This exceeded the required referral chain length to achieve equilibrium, which was estimated at three or four waves of accrual for key study indicators (age, gender identity and sexual orientation) in both cities.

A total of 862 participants were included in this analysis, with 546 from the clinical care site in Abuja and 316 from the Lagos site. The median age of participants was 24 [interquartile range (IQR) 21–27] years. Participants from Abuja were less likely to self-identify as female (9.9% vs. 16.1%, *p*<0.05) and were more likely to self-identify as bisexual (70.5% vs. 56.0%, *p*<0.001). Participants from Abuja were more likely to be Muslim, tended to have a lower level of education, and were more likely to be married or living with a female partner (Table 1).

**Table 1:** Demographic characteristics of the study population stratified by site

	Abuja (*n*=546)		Lagos (*n*=316)		
			
Characteristics	Crude *n* (%)	RDS-weighted % (95% CI)	Crude *n* (%)	RDS-weighted % (95% CI)	*p*
Age					
≤21 years	159 (29.1)	29.8 (24.4–35.1)	113 (35.8)	32.5 (22.2–42.8)	0.640
22–30 years	313 (57.3)	57.7 (51.9–63.4)	181 (57.3)	55.3 (42.5–68.1)	0.741
>30 years	74 (13.6)	12.6 (8.5–16.6)	22 (7.0)	12.2 (0.0–25.8)	0.957
Gender identity					
Male	455 (83.3)	82.4 (77.6–87.2)	243 (76.9)	76.8 (64.4–89.1)	0.403
Female	54 (9.9)	13.7 (9.0–18.5)	51 (16.1)	10.1 (5.1–15.2)	0.313
Other/unknown	37 (6.8)	3.9 (2.1–5.6)	22 (7.0)	13.1 (0.1–26.1)	0.167
Sexual orientation					
Gay/homosexual	158 (28.9)	24.7 (19.9–29.4)	137 (43.4)	38.6 (26.8–50.3)	**0.032**
Bisexual	385 (70.5)	74.6 (69.8–79.3)	177 (56.0)	61.1 (49.4–72.9)	**0.038**
Other/unknown	3 (0.5)	0.8 (0.0–1.8)	2 (0.6)	0.3 (0.0–0.8)	0.392
Religion^a^					
Christian	349 (63.9)	66.4 (60.9–71.9)	273 (86.4)	87.9 (82.1–93.7)	**<0.001**
Muslim	191 (35.0)	33.0 (27.6–38.4)	41 (13.0)	12.1 (6.3–17.9)	**<0.001**
None/other/unknown	6 (1.1)	0.6 (0.0–1.4)	2 (0.6)	–	–
Education level^a^					
Junior secondary or Less	107 (19.6)	17.9 (13.6–22.2)	8 (2.5)	9.4 (0.0–22.5)	0.226
Senior secondary	235 (43.0)	43.1 (37.2–49.1)	209 (66.1)	64.3 (51.9–76.8)	**0.003**
Higher than senior secondary	197 (36.1)	38.4 (32.3–44.4)	99 (31.3)	26.3 (17.2–35.4)	**0.030**
Unknown	7 (1.3)	0.6 (0.0–1.2)	0 (0.0)	–	–
Occupation					
Unemployed	113 (20.7)	20.9 (15.7–26.1)	77 (24.4)	19.6 (10.7–28.6)	0.807
Student	120 (22.0)	24.2 (19.0–29.4)	76 (24.1)	22.1 (14.6–29.6)	0.654
Professional/self-employed	175 (32.1)	29.2 (23.8–34.5)	50 (15.8)	20.4 (11.8–29.0)	0.091
Entertainment/hospitality	65 (11.9)	11.0 (7.3–14.6)	39 (12.3)	9.0 (4.2–13.7)	0.511
Driver/labourer	12 (2.2)	3.0 (0.8–5.3)	9 (2.8)	2.1 (0.3–4.0)	0.537
Other/unknown	61 (11.2)	11.7 (7.2–16.2)	65 (20.6)	26.7 (13.3–40.0)	**0.037**
Marital status					
Single/never married	470 (86.1)	88.0 (84.4–91.6)	282 (89.2)	90.5 (83.9–97.1)	0.520
Married/living with a woman	53 (9.7)	8.7 (5.5–11.9)	8 (2.5)	2.0 (0.0–5.0)	**0.003**
Living with a man	3 (0.5)	0.3 (0.0–0.6)	16 (5.1)	6.1 (0.3–12.0)	0.051
Divorced/widowed/separated/other	20 (3.7)	3.0 (1.4–4.7)	10 (3.2)	1.4 (0.3–2.6)	0.122

RDS, respondent-driven sampling; CI, confidence interval. Adjusted population-level proportions and 95% confidence intervals are weighted for network sizes and recruitment patterns. Adjusted population-level proportions and standard errors were used to calculate a *z*-score and its associated *p*-value. Statistically significant *p*-values (*p*<0.05) are shown in bold.

a RDS-weighting could not be performed for the “none/other/unknown” category of religion and “unknown” category of education level in Lagos due to too few observations. These observations were dropped from the RDS-weighting calculation for other categories of religion and education level, respectively.

Only 169 (19.6%) participants had disclosed their MSM status to family members prior to enrolment, and 316 (36.7%) had disclosed their MSM status to health care providers. In the 12 months prior to enrolment, participants reported having had insertive anal sex with a median of two (IQR 1–5) male partners and receptive anal sex with two (IQR 0–5) partners.

Of the 663 participants reporting any insertive anal sex in the 12 months prior to enrolment, 370 (55.8%) reported condom use “almost always” or “always” during insertive anal sex. Of the 658 participants reporting any receptive anal sex, 311 (47.3%) reported condom use “almost always” or “always” during receptive anal sex. In addition, 71 (10.8%) reported “never” using condoms during receptive anal sex.

### Prevalence of HIV, chlamydia and gonorrhoea

Of the 862 total participants, 473 (54.9%) were HIV seropositive at enrolment. Of these, 275 (58.1%) were previously aware of their status. After adjusting for the RDS recruitment method, HIV prevalence was much lower in Abuja [43.5% (95% CI 37.3–49.6%)] than in Lagos [65.6% (95% CI 54.7–76.5%); Table 2].

**Table 2:** Prevalence of sexually transmitted infections in Abuja and Lagos

	Abuja (*n*=546)		Lagos (*n*=316)	
		
Sexually transmitted infection	Crude % (95% CI)	RDS-weighted % (95% CI)	Crude % (95% CI)	RDS-weighted % (95% CI)
Chlamydia				
Any	14.7 (11.9–17.9)	17.0 (11.8–22.3)	19.3 (15.3–24.0)	18.3 (11.1–25.4)
Urogenital	4.6 (3.1–6.6)	4.3 (2.2–6.5)	5.4 (3.4–8.5)	5.4 (1.8–8.9)
Rectal	11.7 (9.2–14.7)	14.9 (9.9–19.8)	16.5 (12.8–21.0)	15.9 (9.2–22.3)
Gonorrhoea				
Any	20.9 (17.7–24.5)	19.1 (14.6–23.5)	30.1 (25.2–35.4)	25.8 (17.1–34.6)
Urogenital	3.5 (2.2–5.4)	3.85 (1.6–6.1)	4.4 (2.6–7.4)	4.1 (1.3–6.8)
Rectal	19.8 (16.6–23.3)	17.9 (13.7–22.1)	29.4 (24.7–34.7)	25.3 (16.9–33.7)
HIV	46.0 (41.8–50.2)	43.5 (37.3–49.6)	70.3 (65.0–75.1)	65.6 (54.7–76.5)

CI, confidence interval; RDS, respondent-driven sampling. Adjusted population-level proportions and 95% confidence intervals are weighted for network sizes and recruitment patterns.

Across both clinical care sites, the overall prevalence of chlamydia was 16.4%, and the overall prevalence of gonorrhoea was 24.2%. After adjusting for the RDS recruitment methods, the prevalence of chlamydia was 17.0% (95% CI 11.8–22.3%) in Abuja and 18.3% (95% CI 11.1–25.4%) in Lagos. The RDS-weighted prevalence of gonorrhoea was 19.1% (95% CI 14.6–23.5%) in Abuja and 25.8% (95% CI 17.1–34.6%) in Lagos. Overall, 37 (65.6%) participants with gonorrhoea and 73 (51.8%) with chlamydia also had HIV infection, while 61 participants had chlamydia and gonorrhoea co-infection.

The rectum was the predominant anatomic site of infection for both chlamydia and gonorrhoea. Among the 141 participants found to be infected with chlamydia, 99 (70.2%) were infected only at the anorectal site, 25 (17.7%) only in the urogenital tract and 17 (12.1%) at both anatomic sites. Among the 209 participants with gonorrhoea, 176 (84.2%) were infected only at the anorectal site, 8 (3.8%) only in the urogenital tract and 25 (12.0%) at both anatomic sites (Table 3).

**Table 3:** Anatomic sites of gonorrhoea and chlamydia infection amongst study participants

Sexually transmitted infection	N (%)
Chlamydia (*n*=141)	
Urogenital only	25 (17.7%)
Rectal only	99 (70.2%)
Both urogenital and rectal	17 (12.1%)
Gonorrhoea (*n*=209)	
Urogenital only	8 (3.8%)
Rectal only	176 (84.2%)
Both urogenital and rectal	25 (12.0%)

**Figure 1:**
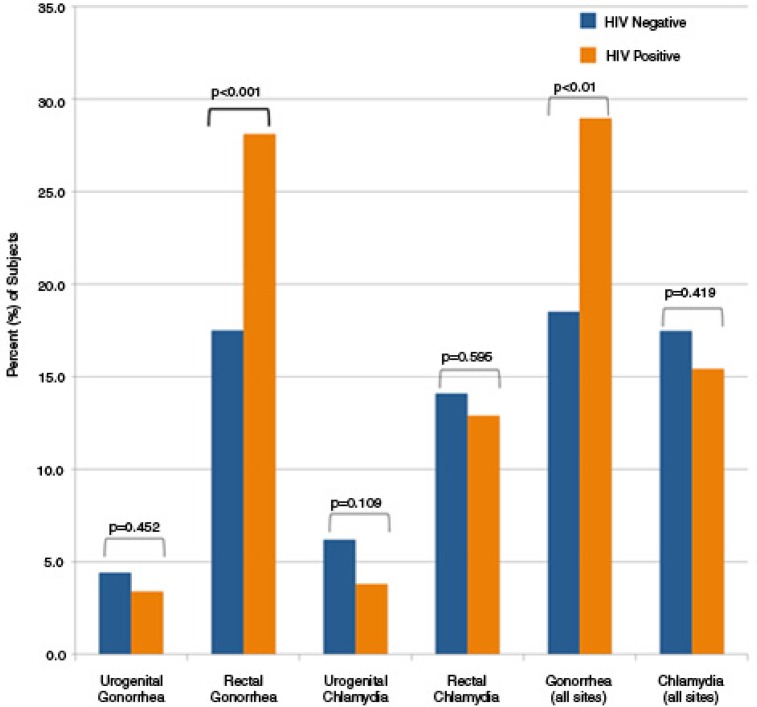
STI prevalence amongst study participants stratified by HIV status.

The prevalence of gonorrhoea was higher among HIV-positive participants than among HIV-negative participants (29.0% vs. 18.5%, *p*<0.001). This relationship was driven solely by an increased prevalence at the rectal site (28.0% of HIV-positive participants vs. 18.0% of HIV-negative participants, *p<*0.001). No difference in prevalence of gonorrhoea at the urogenital site was noted (3.8% of HIV-positive participants vs. 6.2% of HIV-negative, *p>*0.05). Similarly, the prevalence of chlamydia did not differ between HIV-positive (15.4%) and HIV-negative (17.5%) participants at any site (*p>*0.05) (Figure 1).

### Symptoms of chlamydia and gonorrhoea

No symptoms were reported among the 42 participants with urogenital chlamydia or 33 participants with urogenital gonorrhoea. Among the 116 participants with rectal chlamydia, the only symptom reported was a single case of genital/rectal pain (0.9%). Among the 201 participants with rectal gonorrhoea symptoms included one complaint of fever/sweats (0.5%), five complaints of rash (2.6%), two complaints of genital/rectal pain (1.0%) and one complaint of dysuria (0.5%).

### Factors associated with HIV, chlamydia and gonorrhoea

Multivariable analyses of factors associated with HIV and factors associated with chlamydia and/or gonorrhoea are presented in Table 4. Increasing age was associated with a reduced risk of STIs but an increased risk of HIV infection. Increasing number of partners for receptive anal intercourse was associated with an increased risk of both HIV and STI infection, but there was no association between HIV and STI infection and number of insertive anal sex partners. There was a negative association between the number of partners for vaginal intercourse and the prevalence of both HIV and STIs.

**Table 4:** Risk factors for HIV and other sexually transmitted infections

	Chlamydia/gonorrhoea		HIV	
		
Characteristics	Unadjusted risk ratio (95% CI)	Adjusted risk ratio (95% CI)	Unadjusted risk ratio (95% CI)	Adjusted risk ratio (95% CI)
Age				
≤21 years	Reference	Reference	Reference	Reference
22–30 years	**0.81 (0.67–0.98)**	0.87 (0.70–1.07)	**1.44 (1.23–1.68)**	**1.38 (1.18–1.60)**
>30 years	**0.38 (0.24–0.62)**	**0.48 (0.29–0.82)**	**1.40 (1.13–1.74)**	**1.32 (1.06–1.65)**
Gender identity				
Male	Reference	Reference	Reference	Reference
Female	1.24 (0.95–1.60)	1.07 (0.82–1.40)	**1.41 (1.22–1.62)**	1.15 (0.99–1.33)
Other/unknown	1.10 (0.77–1.58)	0.88 (0.61–1.28)	**1.47 (1.25–1.74)**	1.14 (0.98–1.34)
Sexual orientation				
Gay/homosexual	Reference	Reference	Reference	Reference
Bisexual	0.98 (0.81–1.19)	**1.25 (1.00–1.55)**	**0.82 (0.73–0.93)**	1.01 (0.89–1.14)
Other/unknown	1.18 (0.40–3.50)	1.87 (0.72–4.86)	1.29 (0.82–2.02)	1.30 (0.82–2.08)
Religion				
Christian	Reference	Reference	Reference	Reference
Muslim	0.72 (0.57–0.92)	0.78 (0.60–1.00)	**0.74 (0.63–0.87)**	**0.81 (0.69–0.95)**
None/other/unknown	0.34 (0.05–2.15)	0.41 (0.06–2.57)	1.27 (0.85–1.91)	1.19 (0.78–1.81)
Education level				
Junior secondary or Less^a^	Reference	Reference	Reference	Reference
Senior secondary	**1.78 (1.25–2.53)**	**1.52 (1.06–2.17)**	**1.54 (1.21–1.96)**	**1.43 (1.14–1.77)**
Higher than senior secondary	1.33 (0.91–1.93)	1.21 (0.82–1.79)	**1.51 (1.18–1.94)**	**1.35 (1.07–1.70)**
Occupation				
Unemployed	Reference	Reference	Reference	Reference
Student	0.97 (0.76–1.24)	0.95 (0.74–1.22)	**0.73 (0.59–0.89)**	**0.80 (0.66–0.97)**
Professional/self-employed	**0.62 (0.47–0.83)**	0.82 (0.61–1.11)	0.87 (0.74–1.04)	0.93 (0.79–1.09)
Entertainment/hospitality	0.87 (0.63–1.19)	0.94 (0.68–1.29)	0.94 (0.77–1.17)	1.00 (0.83–1.21)
Driver/labourer	0.60 (0.27–1.30)	0.67 (0.30–1.48)	1.05 (0.74–1.50)	1.03 (0.74–1.43)
Other/unknown	0.79 (0.58–1.08)	0.90 (0.66–1.22)	**1.21 (1.03–1.43)**	**1.18 (1.02–1.37)**
Marital status				
Single/never married	Reference	Reference	Reference	Reference
Married/living with a woman	**0.51 (0.30–0.88)**	0.77 (0.43–1.33)	1.04 (0.83–1.32)	1.17 (0.92–1.49)
Living with a man	0.75 (0.35–1.60)	0.69 (0.31–1.52)	**1.48 (1.16–1.89)**	1.16 (0.92–1.48)
Divorced/widowed/ separated/other	0.76 (0.41–1.38)	0.95 (0.55–1.65)	**1.44 (1.17–1.77)**	**1.31 (1.08–1.60)**
Partners for insertive anal sex^b^				
0	Reference	Reference	Reference	Reference
1–2	0.88 (0.68–1.14)	1.10 (0.85–1.43)	0.86 (0.74–1.00)	0.97 (0.84–1.12)
3–9	0.81 (0.63–1.04)	1.01 (0.78–1.32)	**0.71 (0.60–0.83)**	0.88 (0.75–1.02)
≥10	0.99 (0.74–1.31)	1.25 (0.93–1.69)	0.90 (0.76–1.07)	1.09 (0.92–1.31)
Partners for receptive anal sex^b^				
0	Reference	Reference	Reference	Reference
1–2	1.28 (0.93–1.76)	1.18 (0.85–1.64)	**1.73 (1.38–2.16)**	**1.60 (1.29–1.99)**
3–9	**1.66 (1.25–2.21)**	**1.44 (1.07–1.94)**	**1.81 (1.46–2.23)**	**1.70 (1.38–2.10)**
≥10	**1.77 (1.28–2.44)**	**1.50 (1.06–2.13)**	**1.99 (1.58–2.50)**	**1.72 (1.37–2.17)**
Partners for vaginal sex^b^				
0	Reference	Reference	Reference	Reference
1–2	0.85 (0.69–1.06)	0.90 (0.72–1.12)	0.97 (0.86–1.09)	0.99 (0.87–1.13)
3–9	**0.71 (0.53–0.94)**	**0.69 (0.51–0.94)**	**0.45 (0.35–0.58)**	**0.54 (0.42–0.69)**
≥10	0.57 (0.33–1.00)	**0.49 (0.27–0.88)**	**0.41 (0.25–0.67)**	**0.42 (0.26–0.71)**

a Eight participants with unknown education level were included in the reference category.

b Participants were asked to report the number of sexual partners during the 12 months prior to study enrolment.

CI, confidence interval. Univariable log-binomial regression was used to calculate the unadjusted risk ratio for each characteristic of interest. Multivariable log-binomial regression was used to calculate adjusted risk ratios using models that included all the variables in the table. Statistically significant risk ratios (*p*<0.05) are in bold.

## Discussion

This study documented a high burden of infection with HIV, chlamydia and gonorrhoea among highly marginalized populations of MSM and TGW in Nigeria. Importantly, the vast majority of chlamydia and gonorrhoea infections were asymptomatic at the time of diagnosis, and the anorectal site was the most common site of infection. Therefore, these data suggest that syndromic management of STIs is an inadequate strategy for reducing the burden of STIs among MSM and TGW in Nigeria and that routine screening is needed at extragenital sites. Universal screening for asymptomatic STIs that includes anorectal sampling will be required to identify and treat significant reservoirs of disease. Furthermore, this screening must begin at a young age, when risk of STI acquisition is highest.

The RDS-weighted prevalence of HIV among MSM and TGW in Abuja is more than 10 times the reported prevalence of 3.4% in the general Nigerian population [25,26]. The RDS-weighted prevalence of HIV among MSM and TGW in Lagos is even higher. The prevalence rates observed in this study are also considerably higher than the 17.2% prevalence among Nigerian MSM that has been previously reported in the IBBSS [14]. Disproportionately high HIV seroprevalence among MSM has been reported in several other sub-Saharan African countries [2,16,18,19,27,28]. This is suggestive of a significant HIV epidemic in the MSM community at a time when new HIV infections and overall prevalence are declining in the general population [29,30]. Increasing age and increasing number of partners for anal receptive sex were associated with prevalent HIV infection in this study. Conversely, the risk for chlamydia or gonorrhoea decreased with age. This suggests that HIV acquisition in MSM occurs at an early age, before uptake of preventive measures that may limit the risk of STI transmission [31–33]. The similar risk ratios observed for the age groups 22–30 and >30 suggest that new infections are uncommon after the age of 30. Accordingly, the risk from curable STIs, chlamydia and gonorrhoea, is decreased in the older age groups. In other studies, poor perception of HIV infection risk and high mobility especially amongst young MSM were also associated with the increasing risk of HIV infection [34–37]. In response to the multifaceted risks and vulnerabilities that put the MSM community at a greater risk of HIV infection and STIs, risk reduction interventions should employ a comprehensive approach that includes behavioural, biomedical and structural interventions [38,39]. These interventions should be targeted at adolescents and young adults who are at very high risk of HIV infection but have limited access to appropriate prevention services [31,40].

The prevalence of both chlamydia and gonorrhoea is higher in our study than the 4.2% prevalence of both infections among MSM in Lagos reported in an earlier unpublished report that used voluntary screening with urethral swabs or syndromic diagnosis [15]. Universal screening programmes deployed in Tanzania, Botswana and Kenya have reported similarly high STI prevalence rates of between 12 and 20% in a relatively young population [16,17,19]. The high burden of HIV and other STIs among MSM and TGW makes this group an important target for interventions to reduce the global prevalence and incidence of these diseases. To ensure prompt diagnosis, there is a need to better integrate effective STI and HIV laboratory testing and disease management in this population.

There is particularly a high burden of anorectal STIs among the participants in this study, with over 85% of chlamydia infections and 95% of gonorrhoea infections occurring at this anatomic site. This is similar to the over 90% preponderance of rectal STIs in a similar study of MSM in Tanzania [16]. The risk of HIV transmission through anal intercourse is estimated to be 18 times higher than that through vaginal intercourse [41]. This increased risk is due to the higher HIV viral load in rectal secretions compared to semen [42–44]. Anal STIs cause inflammation and increase viral shedding in semen [42]. The current practice of selective testing and management on indication of symptoms and sexual history may not be a sufficient STI control strategy for anorectal STIs. This study showed a low rate of disclosure of MSM status amongst study participants. This lack of disclosure may be due to previous experiences and/or fear of stigma [7,45–47]. Without disclosure, appropriate anorectal screening may not be performed, and these infections would be missed. This underscores the need for MSM-oriented healthcare delivery services, where patients will feel comfortable to disclose their sexual behaviours and will be offered appropriate extragenital screening for STIs.

Many cases of STIs found in this study were asymptomatic and would have been missed by the earlier surveys in Nigeria, which relied on symptomatic review before screening [14,22]. Syndromic management of STIs as currently practiced in the country is inadequate in this population, and there is a need to employ active laboratory-based screening for both symptomatic and asymptomatic persons as the tool of choice for detecting these curable infections.

This study has several strengths. First, use of the RDS recruitment strategy enabled enrolment and characterization of a highly marginalized population of Nigerian MSM and TGW. Second, the standardized questionnaire enabled thorough description of risk behaviours, disclosure to health care providers and other population characteristics. Third, the practice of universal screening for STIs provided a comprehensive assessment of the total burden of disease in this high-risk population. This study also has several limitations. Importantly, it is possible that symptomatic STIs are more likely to have been diagnosed and treated as part of routine care outside of this study, resulting in an overestimate of the proportion of all infections that present asymptomatically. Furthermore, self-reporting of sensitive information and the interviewer’s presence may result in reporting biases, particularly given the stigma surrounding homosexuality in Nigeria. The potentially stigmatizing nature of receiving care at MSM-friendly community health centres and the potential for harassment by authorities or others may have impeded recruitment into this study. This analysis evaluated characteristics at the level of individual participants in the cohort and did not account for relationship status, such as casual versus regular partnership or exchange of sex for goods or money, which could vary across any one participant’s multiple sexual encounters and could influence sexual behaviours that contribute to the risk of HIV and other STIs [48,49]. Finally, the analysis of risk factors for HIV and other STIs used pooled data from both Abuja and Lagos, and the estimation of risk ratios did not incorporate weighting to account for recruitment via RDS, which could have biased some effect estimates. The conclusions drawn from the two large urban populations in this study may not be generalizable to MSM communities elsewhere in Nigeria or in other parts of Africa.

## Conclusions

There is a high burden of infection with HIV and asymptomatic chlamydia and gonorrhoea among MSM and TGW in Nigeria. Most chlamydia and gonorrhoea cases would have been missed without anorectal screening, which might only occur upon disclosure of MSM status to a health care provider. Such disclosure was exceedingly uncommon among participants prior to enrolment in this cohort. MSM-focused, trusted community health centres may be used as a model of care to facilitate this disclosure and provide health care services that meet the unique needs of the MSM and TGW in Nigeria. Taken together, these data suggest the need for interventions to better support Nigerian MSM and TGW for appropriate STI screening and management.

## Prior Presentation

This work was presented, in part, at IDWeek 2015, San Diego, CA, 6–11 October 2015.
